# Variation in a Newly Identified Caprine *KRTAP* Gene Is Associated with Raw Cashmere Fiber Weight in Longdong Cashmere Goats

**DOI:** 10.3390/genes12050625

**Published:** 2021-04-22

**Authors:** Mengli Zhao, Huitong Zhou, Yuzhu Luo, Jiqing Wang, Jiang Hu, Xiu Liu, Shaobin Li, Kaiwen Zhang, Huimin Zhen, Jon G. H. Hickford

**Affiliations:** 1 Gansu Key Laboratory of Herbivorous Animal Biotechnology, College of Animal Science and Technology, Gansu Agricultural University, Lanzhou 730070, China; zhaoml@st.gsau.edu.cn (M.Z.); luoyz@gsau.edu.cn (Y.L.); huj@gsau.edu.cn (J.H.); liux@gsau.edu.cn (X.L.); lisb@gsau.edu.cn (S.L.); zhenhm@st.gsau.edu.cn (H.Z.); 2 International Wool Research Institute, Gansu Agricultural University, Lanzhou 730070, China; huitong.zhou@lincoln.ac.nz; 3 Gene-Marker Laboratory, Faculty of Agriculture and Life Sciences, Lincoln University, Lincoln 7647, New Zealand; 4 Program in Developmental and Stem Cell Biology, Research Institute, The Hospital for Sick Children, University of Toronto, Toronto, ON M5G 0A4, Canada; kw.zhang@mail.utoronto.ca

**Keywords:** *KRTAP1-2*, polymerase chain reaction-single strand conformation polymorphism, cashmere fiber traits, gene expression

## Abstract

Keratin-associated proteins (KAPs) and keratins determine the physical and chemical properties of cashmere fibers as they are the main components of the fibers. It has been reported that ovine *KRTAP1-2* affects clean fleece weight, greasy fleece weight and yield in sheep, but the gene has not been described in goats and its effects on fiber traits are unknown. In this study, we identify the keratin-associated protein 1-2 gene (*KRTAP1-2*) in the goat genome and describe its effect on cashmere fiber traits in 359 Longdong cashmere goats. Six sequence variants (named CAPHI-KRTAP1-2*A to CAPHI-KRTAP1-2*F) were revealed using polymerase chain reaction-single strand conformation polymorphism (PCR-SSCP) analysis. These sequences have the highest homology with ovine *KRTAP1-2* sequences. There were a 60-bp deletion, a 15-bp insertion and five single nucleotide polymorphisms (SNPs) including two non-synonymous SNPs in the coding sequence. The caprine *KRTAP1-2* gene was expressed in the skin tissue, but a signal was not observed for the kidneys, liver, lungs, spleen, heart and *longissimus dorsi* muscle. Variation in caprine *KRTAP1-2* was found to be associated with raw cashmere fiber weight, but not with fiber diameter and length.

## 1. Introduction

The Longdong cashmere goat is a special breed of goat farmed in the Longdong area of the Gansu Province. This breed has been created as a cross between the Ziwuling black goat, the Inner Mongolian cashmere goat and the Liaoning cashmere goat (Supplementary Materials Figure S1). While the Longdong cashmere goat is well-adapted to harsh environments including desert and other arid regions, it has lower cashmere fiber yields (average of 400 g per annum) than the Liaoning cashmere goat (average of 640 g) and the Inner Mongolian cashmere goat (average of 450 g). The identification and understanding of genes that regulate cashmere fiber growth and structure is therefore important for improving the yield of cashmere fiber in Longdong cashmere goats.

Cashmere fibers are produced by secondary hair follicles and the fibers are characterized as being soft, elastic and strong, and they provide good thermal insulation. As with wool and hair, the most common protein structural components of cashmere fibers are keratins and keratin association proteins (KAPs), with the latter serving as a matrix that cross-links with the keratins via disulfide bond formation [1].

The KAPs have historically been classified into three categories depending on their content of the amino acids-cysteine, or glycine/tyrosine [2]. They are typically encoded by small intron-less genes called *KRTAPs*, and over 100 *KRTAPs* from 29 families have been identified across mammalian species [2,3,4]. Over the last four decades, the identification of *KRTAPs* and research into the effect of these genes on hair and wool traits has been most commonly focused on humans and sheep. However, to date, only 17 *KRTAPs* have been identified from 13 families in goats [5,6,7,8,9].

The genetic similarity between sheep and goats suggests that many *KRTAPs* remain to be identified in the goat genome. Of the 17 caprine *KRTAPs* identified to date, seven of them have been reported to affect cashmere fiber length, cashmere fiber yield and cashmere mean fiber diameter, including *KRTAP8-2* [10], *KRTAP20-2* [11], *KRTAP20-1* [12], *KRTAP15-1* [6], *KRTAP27-1* [9], *KRTAP28-1* [8] and *KRTAP24-1* [7]. This suggests that nucleotide sequence variation in the caprine *KRTAPs* is an important determinant of key cashmere traits.

The KAP1 proteins are encoded by a gene family that is well characterized in sheep, with four variable ovine *KRTAP1s* identified: *KRTAP1-1*, *KRTAP1-2*, *KRTAP1-3* and *KRTAP1-4* [13,14,15]. The ovine *KRTAP1s* are located on ovine chromosome 11, and in proximity to where quantitative trait locus (QTLs) for wool weight and staple strength have been found [16]. The ‘QTSCCQPXXX’ decapeptide repeat in the N-terminal region is a common characteristic of the ovine KAP1 family [2,15,17], and the family displays a strong co-evolutionary pattern within and between species [18].

Of the KAP1 genes, ovine *KRTAP1-2* is expressed in the cortex of the wool fiber [15,19]. Eleven variants and 10 single nucleotide polymorphisms (SNPs) have been detected for ovine *KRTAP1-2*, and the presence of some of these has been associated with wool yield, greasy fleece weight and clean fleece weight [19]. Taken together, the evidence suggests that ovine *KRTAP1-2* variation may allow improvement in wool production, and hence it might be speculated that caprine *KRTAP1-2* may be important for fiber production in goats too.

The aim of this study was to identify caprine *KRTAP1-2* and to assess the relationship between variation in the gene and fiber traits in Longdong cashmere goats. The expression of *KRTAP1-2* in different caprine tissues was also investigated. This study may provide new insight into improving fiber traits for Longdong cashmere goats.

## 2. Materials and Methods

### 2.1. Cashmere Fiber, Blood and Tissue Collection

Three hundred and fifty-nine one-year-old Longdong cashmere goats from 11 unrelated sires were selected, with these being reared at the Yusheng Cashmere Goat Breeding Company in Huan County of the Gansu Province. The raw cashmere fiber weight was measured after it had been collected by combing. Small samples of the fibers were also collected from the mid-side region of each goat’s body, and the cashmere fiber length and mean fiber diameter (MFD) were tested using the Optical Fiber Length and Diameter Analyzer OFDA4000 (EPCO, Shanghai, China) platform.

Additionally, three separate female twelve-month-old Longdong cashmere goats were slaughtered to collect tissue from the skin, kidneys, liver, lungs, spleen, heart, and *longissimus dorsi* muscle. The tissue samples were frozen and stored in liquid nitrogen prior to reverse-transcription PCR (RT-PCR) analysis.

Blood samples from these goats were collected onto FTA cards (Whatman BioScience, Middlesex, UK) and genomic DNA samples were prepared for subsequent analyses using a two-step washing procedure [20].

### 2.2. Screening for Sequence Polymorphism in Caprine KRTAP1-2

A Basic Local Alignment Search Tool (BLASTN) search (https://blast.ncbi.nlm.nih.gov/Blast.cgi?PAGE_TYPE=BlastSearch; accessed on 15 November 2020) was undertaken of Caprine Genome Assembly ASM170441v1 using the ovine *KRTAP1-2* coding sequence (GenBank sequence HQ897973). The sequence with the highest homology to ovine *KRTAP1-2* sequence was hypothesized to be the putative caprine *KRTAP1-2* sequence.

Polymerase Chain Reaction (PCR) primers (5’-TAACAACCCTCCTCTCAATCT-3’ and 5’-TTCATGGACTGAAGTTGAACT-3’) were designed to amplify the putative caprine *KRTAP1-2* from this sequence. Amplifications were undertaken in 20-µL reactions, which included the purified genomic DNA on a 1.2-mm punch of the FTA paper, Mg^2+^ (2.5 mM), Taq DNA polymerase (0.5U, Takara, Dalian, China), deoxyribonucleoside triphosphates (dNTPs) (150 µM, Takara, Dalian, China), the primers (0.25 µM), 10 × PCR buffer supplied with DNA polymerase enzyme (2.0 µL) and deionized water (dH_2_O) to volume. The amplifications were carried out in Bio-Rad S1000 thermal cyclers (Bio-Rad, Hercules, CA, USA) and consisted of 5 min at 94 °C for an initial denaturation, followed by 35 cycles of 30 s at 94 °C, 30 s at 53 °C, 30 s at 72 °C, and lastly 5 min at 72 °C for a final extension. There was a test for PCR product amplification in 1 × TBE buffer using 1.0% agarose gels electrophoresis.

The PCR amplicons were subjected to single strand conformation polymorphism (SSCP) analysis. One microliter aliquots of the PCR amplification products were added to 8.0 µL aliquots of loading dye (98% formamide, 0.025% bromophenol blue, 0.025% xylene cyanol and 10 mM ethylenediaminetetraacetic acid (EDTA) and then denatured for 10 min at 95 °C. The mixtures were cooled on wet ice and then loaded on 16 cm × 18 cm, 12% acrylamide: bisacrylamide (37.5:1) (Bio-Rad, Hercules, CA, USA) gel. Electrophoresis was carried out at 180 V for 23 h at 31.5 °C in 0.5 × TBE buffer using Protean II xi cells (Bio-Rad). After electrophoresis, the gels were stained to identify the DNA banding patterns using a method described by Byun et al. [21].

Amplicons that produced different SSCP patterns were then selected for DNA sequencing. Those amplicons that appeared to be homozygous according to SSCP analysis were directly sequenced in both directions at the Beijing Genomics Institute (Beijing, China), and those variants that were found only in a heterozygote form were prepared by the method of Gong et al. [22] and then sequenced in both directions at the Beijing Genomics Institute.

The DNAMAN (Lynnon BioSoft, Vaudreuil, QC, Canada) software (version 5.2.10) and ClustalW algorithm was used to align, translate and compare DNA sequences. A phylogenetic tree was built based on the predicted amino acid sequence using MEGA version 7.0 and a maximum-likelihood method.

### 2.3. Reverse Transcription-Polymerase Chain Reaction (RT-PCR)

Total RNA from the seven tissues isolated from the Longdong cashmere goats was extracted with Trizol (Invitrogen, Carlsbad, CA, USA). Spectrophotometry (ultraviolet range) and 2% agarose gels electrophoresis were then used to detect the concentration and determine quality of RNA, respectively.

The PrimeScript RT Reagent Kit with gDNA Eraser (Perfect Real Time, Takara, Dalian, China) was utilized for reverse transcription (RT) of the isolated total RNA to produce cDNA. Next, a PCR amplification with the cDNA as a template and the primers (5’-GTAGCAGCGGAGCTGTGAG-3’ and 5’-CAGGACTGTCCACAGTAGGATG-3’) was used to produce a 170-bp fragment from within the coding sequences of caprine *KRTAP1-2*. The caprine β actin gene (*ACTB*) was used as an internal reference standard in these analyses, with the primers of 5’-AGCCTTCCTTCCTGGGCATGGA-3’ and 5’-GGACAGCACCGTGTTGGCGTAGA-3’ being used to amplify a fragment of this gene. The amplification conditions used were the same as for the genomic amplifications of *KRTAP1-2* described above, but the genomic DNA was substituted with cDNA. The RT-PCR products from three female goats were then analyzed by agarose gels electrophoresis (1% *w/v* gels) to ascertain the presence and quality of the RT-PCR products in order to detect the expression of the gene in different tissues.

### 2.4. Statistical Analyses

General linear mixed-effect models (GLMMs) were used to assess the effect of variation in caprine *KRTAP1-2* on cashmere fiber traits using IBM SPSS Statistics version 24.0 (IBM, Armonk, NY, USA). Single-variant models were firstly used to assess the effect of the absence or presence of individual caprine *KRTAP1-2* variants on variation in the fiber traits. Based on these models, multi-variant models were then employed, with these analyzing the effect of the absence or presence of individual caprine *KRTAP1-2* variants (but with them being corrected for other variants that had *P* < 0.10 and that were therefore potentially affecting the trait). To confirm the variant absence or presence results from the multi-variant models, genotype comparisons were also carried out, with a Bonferroni correction being applied to reduce the probability of false positive results during the multiple comparisons in these models. Gender and sire were included in the GLMMs as a fixed and random factor, respectively, as they affected all the fiber traits (*P* < 0.05). Birth rank was excluded from the models as it did not affect the cashmere traits. Only the main effects were detected.

## 3. Results

### 3.1. Identification of Caprine KRTAP1-2

**Figure 1:**
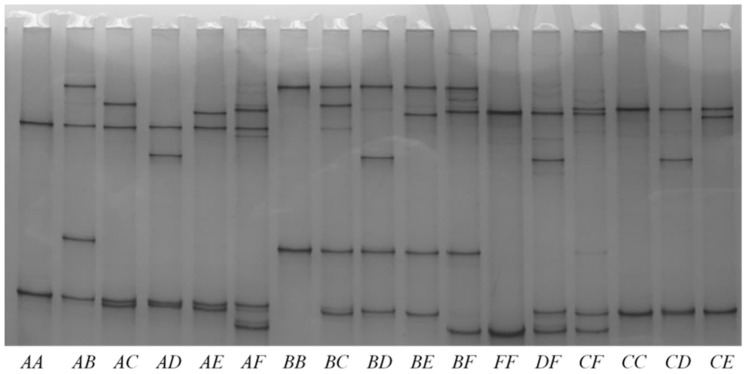
Polymorphism of caprine *KRTAP1-2* identified using PCR-SSCP analysis. Six variants (*A*–*F*) represented by six unique banding patterns were found in arrangements that appeared to correspond to either heterozygous or homozygous genotypes.

**Figure 2:**
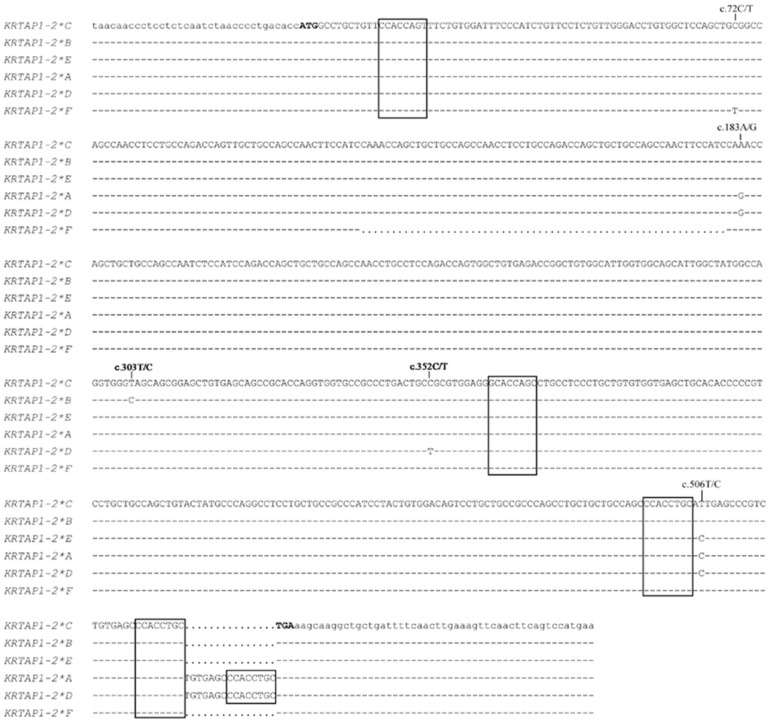
Nucleotide sequence alignment of the caprine *KRTAP1-2* sequences. The dashes indicate the same nucleotide with respect to the sequence of caprine KRTAP1-2*C and the dots represent nucleotide deletions. The single nucleotide polymorphisms identified among these sequences are indicated above the sequences. The notional ATG (start codon) and TGA (stop codon) are shown in bold, and the Chi-like sequences are shown in box. The uppercase letters indicate nucleotides in the coding region, whereas the lowercase letters indicate nucleotides in the non-coding region. The numbering of nucleotides and amino acid positions follows the Human Genome Variation Society (HGVS) nomenclature (http://varnomen.hgvs.org/; accessed on 15 November 2020).

A BLASTN search using the ovine *KRTAP1-2* coding sequence (HQ897973) revealed a highly similar sequence on the goat genome assembly sequence for chromosome 19 (NC_030826.1: nt 40916023 to 40916556). Subsequently, SSCP analysis of PCR amplicons amplified from this gene region revealed six different banding patterns (*A* to *F*) (Figure 1), and upon DNA sequencing, these were found to correspond to six unique DNA sequences, ranging in size from 557-bp to 617-bp (Figure 2).

**Figure 3:**
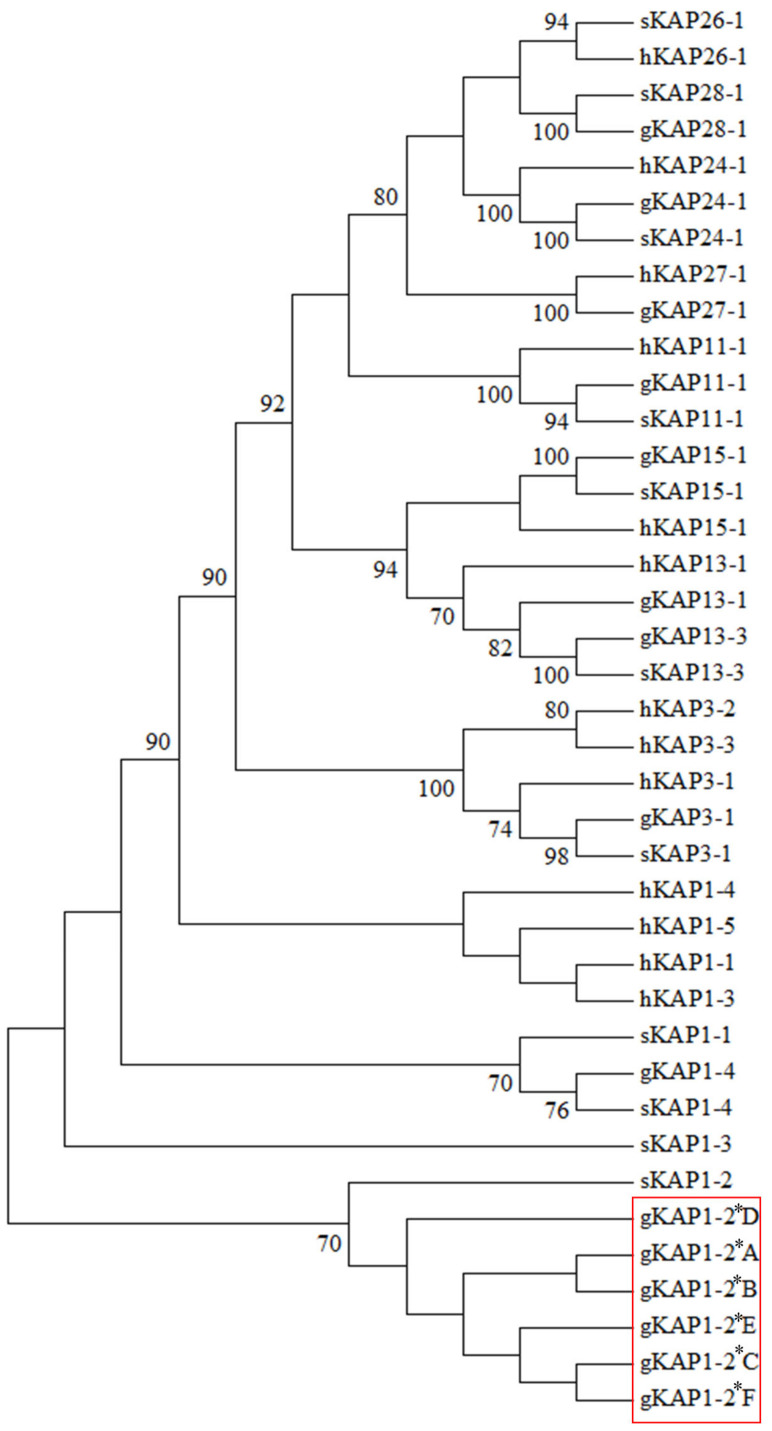
Phylogenetic tree of HS-KAPs identified in goats, humans and sheep. The putative amino acid sequences were used to construct the tree. The prefix “g” indicates the sequences from goats, while prefix “s” and “h” represent sequences from sheep and humans, respectively. The newly identified caprine KAP1-2 sequences are marked in a red box. The numbers at the forks represented the bootstrap confidence values with only those ≥ 70% being shown. The amino acid sequence of caprine KAP28-1 was adopted from Wang et al. [8]. The GenBank accession numbers for the other KAP sequences are: gKAP27-1 (MN934937), gKAP24-1 (MG996011), gKAP15-1 (NM_001285771), gKAP13-3 (JX426138), gKAP13-1 (AY510115), gKAP11-1 (NM_001285767.1), gKAP3-1 (NM_001285774), gKAP1-4 (JN012101.1), sKAP28-1 (MN053920), sKAP26-1 (KX644903), sKAP24-1 (JX112014), sKAP15-1 (MH742372), sKAP13-3 (JN377429), sKAP11-1 (HQ595352), sKAP3-1 (M21099), sKAP1-4 (GQ507741.1), sKAP1-3 (NM_001159761), sKAP1-2 (HQ897975), sKAP1-1 (X01610), hKAP27-1 (AB096937.1), hKAP26-1 (NM_203405.1), hKAP24-1 (NM_001085455.2), hKAP15-1 (NM_181623.2), hKAP13-1 (NM_181599.2), hKAP11-1 (NM_175858.2), hKAP3-3 (NM_033185.2), hKAP3-2 (NM_031959.2), hKAP3-1 (NM_031958.1), hKAP1-5 (NM_031957.1), hKAP1-4 (NM_001257305.1), hKAP1-3 (NM_030966.1) and hKAP1-1 (NM_030967.2).

In the 359 cashmere goats investigated, the frequencies of variants *A*, *B*, *C*, *D*, *E* and *F* were 28.13%, 24.65%, 38.86%, 1.53%, 2.51% and 4.32%, respectively. The frequencies of six common genotypes were 8.91%, 13.93%, 20.61%, 7.24%, 16.71% and 16.43% for *AA*, *AB*, *AC*, *BB*, *BC* and *CC*. Phylogenetic analysis suggested that the amino acid sequence predicted from the goat genome sequence identified was closer to sheep KAP1-2 than all of other HS-KAPs (Figure 3), suggesting that this was the caprine KAP1-2 gene, and also suggesting that the six sequences found are variants of caprine *KRTAP1-2*.

**Figure 4:**
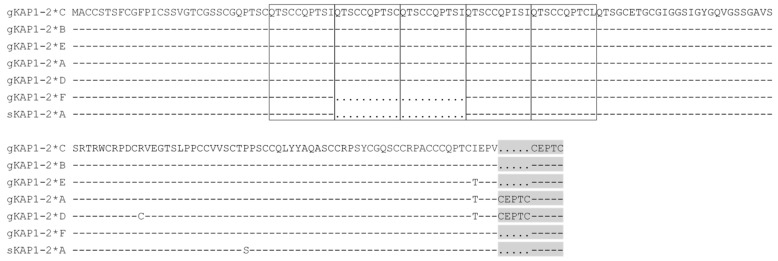
Alignment of the predicted amino acid sequences derived from the caprine KAP1-2 gene sequences and ovine KAP1-2 variant *A*. The prefix “g” and “s” stand for goat and sheep sequences, respectively. The dashes indicate identity with caprine KAP1-2*C and the dots represent amino acid deletions. The decapeptide ‘QTSCCQPT(S/C)X’ repeats are boxed, and pentapeptide ‘CEPTC’ repeats are shaded. The levels of cysteine (21.7–23.1%) and serine (15.3–15.8%) were high, while the content of threonine (9.6–11.5%), proline (9.6–9.9%), glutamine (8.3–9.6%) and glycine (8.2–9.6%) was moderate in the predicted polypeptide sequences encoded by caprine *KRTAP1-2*.

Based on the nomenclature suggested by Gong et al. [23], the *KRTAP1-2* variants were named CAPHI-KRTAP1-2*A to CAPHI-KRTAP1-2*F. Of these, CAPHI-KRTAP1-2*C was identical to the Caprine Genome Assembly sequence (NC_030826.1: nt 40916023 to 40916556). Five Chi-like sequences were found in the caprine *KRTAP1-2* sequences (Figure 2) and five SNPs were identified across the caprine *KRTAP1-2* sequences. All of the SNPs were located within the coding region and these were c.72C/T, c.183A/G, c.303T/C, c.352C/T and c.506T/C (Figure 2). Two SNPs (c.352C/T and c.506T/C) were non-synonymous and would result in the putative amino acid changes of p.Arg118Cys and p.Ile169Thr, respectively (Figure 4).

Aside from the SNPs, a 60-bp deletion and a 15-bp insert were also found for caprine *KRTAP1-2* (Figure 2). The 60-bp deletion was located in a decapeptide repeat coding region and would result in three or five decapeptide repeats, i.e., multiples of QTSCCQPT(S/C)X in the middle region of the protein (Figure 4). The 15-bp insert was located in the repeat region upstream of the stop codon and would lead to one repeat of the pentapeptide (CEPTC) in some sequences and two repeats in the other sequences (Figure 4).

### 3.2. Expression of Caprine KRTAP1-2 in Different Tissues

**Figure 5:**
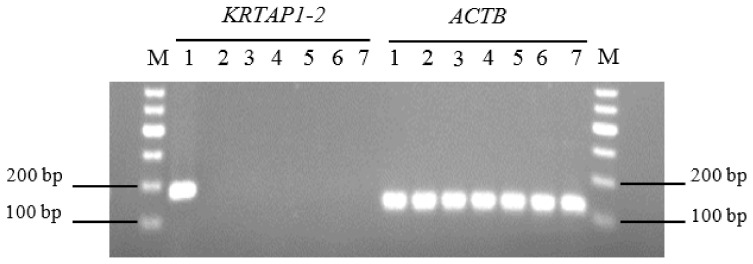
RT-PCR results for caprine *KRTAP1-2* and β actin (*ACTB*) mRNA presence in seven tissues of Longdong cashmere goats. The results obtained from three different goats were identical, so only the results from one of the goats is shown. M: DNA marker; 1: skin; 2: kidney; 3: liver; 4: lung; 5: spleen; 6: heart; and 7: longissimus dorsi muscle.

The RT-PCR analysis revealed that caprine *KRTAP1-2* only appeared to be expressed in the skin, but not in the kidneys, liver, lungs, spleen, heart, and *longissimus dorsi* muscle in all of the three Longdong cashmere goats tested, with the results from one of these goats being shown in Figure 5.

### 3.3. Effect of Variation in Cashmere KRTAP1-2 on Three Cashmere Fiber Traits

Of the six *KRTAP1-2* gene sequences identified in Longdong cashmere goats, only three (CAPHI-KRTAP1-2*A, CAPHI-KRTAP1-2*B and CAPHI-KRTAP1-2*C) occurred at a frequency of over 5%, and hence, associations were only analyzed for these. The presence of CAPHI-KRTAP1-2*B was found to be associated with a decreased cashmere fiber yield in the single-variant model and the association persisted in the multi-variant model when correcting for the effect of the other variants (Table 1). No associations were detected between variation in *KRTAP1-2* and cashmere fiber diameter and length (Table 1). Goats with genotype *BB* produced less cashmere fibers than goats with other common genotypes (Table 2).

## 4. Discussion

This study has identified a new caprine *KRTAP* and describes variation in this gene including the presence of SNPs, insertions and deletions. Some of the sequence variation detected was found to affect the cashmere fiber yield in Longdong cashmere goats. The gene appeared to only be expressed in goat skin tissue, albeit only six other tissues were tested and with a qualitative reverse transcription-polymerase chain reaction approach. We believe other tissues should be tested in future, especially follicle tissue, and with a quantitative RNA assay.

The newly identified caprine *KRTAP* sequences were phylogenetically closest to ovine *KRTAP1-2* and was located in the same chromosomal region as caprine *KRTAP1-3* and *KRTAP1-4*, suggesting that these new *KRTAP* sequences represent variants of caprine *KRTAP1-2*.

The presence of multiple sequences of caprine *KRTAP1-2* is consistent with the previous findings that all of the known *KRTAPs* are polymorphic [2,24]. The inserts/deletions detected for caprine *KRTAP1-2* were associated with repeat regions and lead to variation in the number of repeats. This phenomenon has been described for some other *KRTAPs*, including *KRTAP1-1*, *KRTAP5-4* and *KRTAP6-5* in sheep [17,25,26] and *KRTAP9-2* in goats [27], but it has not been detected for the *KRTAP1-2* orthologue in sheep [2,24]. The presence of three or five QTSCCQPT(S/C)X repeats in the middle region of the putative protein, and one or two CEPTC repeats at the carboxyl-terminus, are similar to the three QTSCCQPT(S/C)X repeats and one CEPTC repeat in ovine KAP1-2, although they may have different functional effects in goats when compared to sheep. Providing evidence of this would, however, require considerably more investigation.

Caution is also needed in comparing the number and type of SNPs identified in these Longdong cashmere goats with sheep. While the number of SNPs found here appears to be less than has been described in the sheep orthologue, the goat SNPs were found in only 359 goats from one breed and one farm, whereas the sheep SNPs reported were discovered in larger numbers of sheep from variety of breeds and from different farms [15,19]. It is therefore reasonable to expect that more SNPs may be identified if more goats from more breeds and more farms are investigated. The SNPs identified in the two species are also located at different positions. This, together with length variation being present in caprine *KRTAP1-2* but absent in ovine *KRTAP1-2*, suggests that different selection pressures may have acted upon sheep and cashmere goats.

There is literature reporting Chi or Chi-like sequences in *KRTAPs* from sheep and goats, including as ovine *KRTAP1-n* [17], ovine *KRTAP15-1* [28], caprine *KRTAP15-1* [6], caprine *KRTAP24-1* [7] and caprine *KRTAP27-1* [9]. Chi (crossover hotspot instigator, χ) is an octamer motif (5’-GCTGGTGG-3’) that has been described as a recombination hotspot in E. coli and related to the recombination of double-strand DNA breaks sites [29]. These Chi-like sequences detected in caprine *KRTAP1-2* may therefore have contributed, at least in part, to the origin of sequence diversity in the gene.

There is only one SNP difference between CAPHI-KRTAP1-2*B and CAPHI-*KRTAP1-*2*C and that SNP was synonymous. The association with cashmere yield was detected for CAPHI-KRTAP1-2*B, but not for CAPHI-*KRTAP1-*2*C, suggesting that this synonymous SNP may either directly have a functional effect, or be linked to another region of the gene that has a functional effect. While synonymous SNPs do not lead to amino acid changes, they have been reported to at times regulate gene function by affecting mRNA secondary structure [30], mRNA stability [31] and the miRNA-based regulation of expression [32]. It is also possible that the effect detected for CAPHI-*KRTAP1-*2*B may be due to the fact that the SNP is linked to other functional SNPs upstream or downstream of the region investigated here, or located in other nearby *KRTAPs*.

The finding that CAPHI-*KRTAP1-*2*B of caprine *KRTAP1-2* was associated with decreased cashmere fiber yield but had no effect on fiber diameter or length, possibly suggests that the presence of *B* may lead to there being a lower number of secondary wool follicles, and hence, less cashmere fibers would be produced. This effect appears to be similar to that reported for its ovine orthologue in which variation in *KRTAP1-2* was found to affect wool fiber weight traits, but not fiber diameter-associated traits and fiber length [19]. The results from this study suggest that breeding against CAPHI-*KRTAP1-*2*B would lead to a high cashmere fiber yield without compromising the fiber diameter, potentially providing a gene marker for improving cashmere fiber production.

## Figures and Tables

**Table 1:** Associations between caprine *KRTAP1-2* sequences and cashmere fiber traits (Mean ± SE) ^1^.

Cashmere Trait	Variant Assessed	Other Variants Fitted	Absent		Present		*P* Value
			Mean ± SE	*n*	Mean ± SE	*n*	
Raw cashmere fiber weight (g)	*A*	None	407.1 ± 4.12	145	416.0 ± 4.05	156	0.075
	*B*	None	**419.5 ± 3.73**	**165**	**400.0 ± 4.29**	**136**	**<0.001**
	*C*	None	405.0 ± 4.80	108	414.6 ± 3.61	193	0.063
	*A*	*B, C*	405.6 ± 4.71	145	412.2 ± 4.17	156	0.264
	*B*	*A, C*	**416.7 ± 4.52**	**165**	**401.1 ± 4.41**	**136**	**0.009**
	*C*	*A, B*	405.9 ± 5.19	108	411.9 ± 3.91	193	0.345
Mean fiber diameter (µm)	*A*	None	13.5 ± 0.04	145	13.6± 0.04	156	0.442
	*B*	None	13.7 ± 0.04	165	13.6 ± 0.04	136	0.977
	*C*	None	13.6 ± 0.04	108	13.6 ± 0.033	193	0.357
Cashmere fiber length (cm)	*A*	None	4.1 ± 0.04	145	4.2 ± 0.04	156	0.099
	*B*	None	4.2 ± 0.04	165	4.2 ± 0.05	136	0.373
	*C*	None	4.2 ± 0.05	108	4.2 ± 0.04	193	0.395

^1^ Estimated standard errors (SEs) and marginal means in general linear mixed effect model, with *P* < 0.05 being shown in bold.

**Table 2:** Association between caprine *KRTAP1-2* genotypes and cashmere fiber traits.

Genotype	Mean ± SE ^1^		
	Raw Cashmere Fiber Weight (g)	Cashmere Fiber Length (cm)	Mean Fiber Diameter (µm)
*AA* (*n* = 32)	422.8 ± 7.53 ^a^	4.3 ± 0.08	13.6 ± 0.07
*AB* (*n* = 50)	408.6 ± 6.16 ^a^	4.2 ± 0.07	13.6 ± 0.06
*AC* (*n* = 74)	413.9 ± 5.09 ^a^	4.2 ± 0.06	13.6 ± 0.05
*BB* (*n* = 26)	369.6 ± 8.49 ^b^	4.1 ± 0.10	13.7 ± 0.08
*BC* (*n* = 60)	403.4 ± 5.66 ^a^	4.1 ± 0.06	13.5 ± 0.05
*CC* (*n* = 59)	423.1 ± 5.55 ^a^	4.2 ± 0.06	13.5 ± 0.05
*P* value	<0.001	0.165	0.326

^1^ Estimated standard errors (SEs) and marginal means in general linear mixed effect model. Means that do not share a superscript letter (a or b) are significantly different at *P* < 0.01.

## Data Availability

The data presented in this study are available on request from the corresponding author.

## Supplementary Materials

The following is available online at: https://www.mdpi.com/article/10.3390/genes12050625/s1, Supplementary Figure S1. Adult and juvenile Longdong cashmere goats.

## References

[1] Powell B.C., Rogers G.E. (1997). The role of keratin proteins and their genes in the growth, structure and properties of hair. EXS.

[2] Gong H., Zhou H., Forrest R.H., Li S., Wang J., Dyer J.M., Luo Y., Hickford J.G.H. (2016). Wool keratin-associated protein genes in sheep-a review. Genes.

[3] Bai L., Wang J., Zhou H., Gong H., Tao J., Hickford J.G.H. (2019). Identification of ovine *KRTAP28-1* and its association with wool weight and mean fibre diameter-associated traits. Animals.

[4] Gong H., Zhou H., Wang J., Li S., Luo Y., Hickford J.G.H. (2019). Characterisation of an ovine keratin associated protein (KAP) gene, which would produce a protein rich in glycine and tyrosine, but lacking in cysteine. Genes.

[5] Parris D., Swart L.S. (1975). Studies on the high-sulphur proteins of reduced mohair. The isolation and amino acid sequence of protein scmkb-m1.2. Biochem. J..

[6] Zhao M., Zhou H., Hickford J.G.H., Gong H., Wang J., Hu J., Liu X., Li S., Hao Z., Luo Y. (2019). Variation in the caprine keratin-associated protein 15-1 (KAP15-1) gene affects cashmere fibre diameter. Arch. Anim. Breed..

[7] Wang J., Zhou H., Luo Y., Zhao M., Gong H., Hao Z., Hu J., Hickford J.G.H. (2019). Variation in the caprine KAP24-1 gene affects cashmere fibre diameter. Animals.

[8] Wang J., Zhou H., Hickford J.G.H., Zhao M., Gong H., Hao Z., Shen J., Hu J., Liu X., Li S. (2020). Identification of caprine *KRTAP28-1* and its effect on cashmere fiber diameter. Genes.

[9] Zhao M., Zhou H., Luo Y., Wang J., Hu J., Liu X., Li S., Hao Z., Jin X., Song Y. (2020). Variation in the caprine keratin-associated protein 27-1 gene is associated with cashmere fiber diameter. Genes.

[10] Liu H., Li N., Jia C., Zhu X., Jia Z. (2007). Effect of the polymorphisms of keratin associated protein 8.2 gene on fibre traits in Inner Mongolia cashmere goats. Asian Australas. J. Anim. Sci..

[11] Wang J., Che L., Hickford J.G.H., Zhou H., Hao Z., Luo Y., Hu J., Liu X., Li S. (2017). Identification of the caprine keratin-associated protein 20-2 (KAP20-2) gene and its effect on cashmere traits. Genes.

[12] Wang J., Hao Z., Zhou H., Luo Y., Hu J., Liu X., Li S., Hickford J.G.H. (2018). A keratin-associated protein (KAP) gene that is associated with variation in cashmere goat fleece weight. Small Rumin. Res..

[13] Itenge-Mweza T.O., Forrest R.H.J., Mckenzie G.W., Hogan A., Abbott J., Amoafo O., Hickford J.G.H. (2007). Polymorphism of the KAP1.1, KAP1.3 and K33 genes in Merino sheep. Mol. Cell. Probes.

[14] Gong H., Zhou H., Hickford J.G.H. (2010). Polymorphism of the ovine keratin-associated protein 1-4 gene (*KRTAP1-4*). Mol. Biol. Rep..

[15] Gong H., Zhou H., Yu Z., Dyer J.M., Plowman J.E., Hickford J.G.H. (2011). Identification of the ovine keratin-associated protein KAP1-2 gene (*KRTAP1-2*). Exp. Dermatol..

[16] Roldan D.L., Dodero A.M., Bidinost F., Taddeo H.R., Allain D., Poli M.A., Elsen J.M. (2010). Merino sheep: A further look at quantitative trait loci for wool production. Animal.

[17] Rogers G.R., Hickford J.G.H., Bickerstaffe R. (1994). Polymorphism in two genes for B2 high sulfur proteins of wool. Anim. Genet..

[18] Zhou H., Visnovska T., Gong H., Schmeier S., Hickford J.G.H., Ganley A.R.D. (2019). Contrasting patterns of coding and flanking region evolution in mammalian keratin associated protein-1 genes. Mol. Phylogenet. Evol..

[19] Gong H., Zhou H., Hodge S., Dyer J.M., Hickford J.G.H. (2015). Association of wool traits with variation in the ovine KAP1-2 gene in Merino cross lambs. Small Rumin. Res..

[20] Zhou H., Hickford J.G.H., Fang Q. (2006). A two-step procedure for extracting genomic DNA from dried blood spots on filter paper for polymerase chain reaction amplification. Anal. Biochem..

[21] Byun S.O., Fang Q., Zhou H., Hickford J.G.H. (2009). An effective method for silver-staining DNA in large numbers of polyacrylamide gels. Anal. Biochem..

[22] Gong H., Zhou H., Hickford J.G.H. (2011). Diversity of the glycine/tyrosine-rich keratin-associated protein 6 gene (KAP6) family in sheep. Mol. Biol. Rep..

[23] Gong H., Zhou H., McKenzie G.W., Yu Z., Clerens S., Dyer J.M., Plowman J.E., Wright M.W., Arora R., Bawden C.S. (2012). An updated nomenclature for keratin-associated proteins (KAPs). Int. J. Biol. Sci..

[24] Gong H., Zhou H., McKenzie G.W., Hickford J.G.H., Luo Y., Clerens S., Dyer J.M., Plowman J.E. (2010). Emerging issues with the current keratin-associated protein nomenclature. Int. J. Trichol..

[25] Gong H., Zhou H., Plowman J.E., Dyer J.M., Hickford J.G.H. (2010). Analysis of variation in the ovine ultra-high sulphur keratin-associated protein KAP5-4 gene using PCR-SSCP technique. Electrophoresis.

[26] Zhou H., Gong H., Wang J., Dyer J.M., Luo Y., Hickford J.G.H. (2016). Identification of four new gene members of the KAP6 gene family in sheep. Sci. Rep..

[27] Yu H., Wang X., Chen H., Wang M., Zhao M., Lan X., Lei C., Wang K., Lai X., Wang X. (2008). The polymorphism of a novel 30bp-deletion mutation at KAP9.2 locus in the cashmere goat. Small Rumin. Res..

[28] Wang J., Zhou H., Zhu J., Hu J., Liu X., Li S., Luo Y., Hickford J.G.H. (2017). Identification of the ovine keratin-associated protein 15-1 gene (*KRTAP15-1*) and genetic variation in its coding sequence. Small Rumin. Res..

[29] Smith G.R. (2001). Homologous recombination near and far from DNA breaks: Alternative roles and contrasting views. Annu. Rev. Genet..

[30] Nackley A.G., Shabalina S.A., Tchivileva I.E., Satterfield K., Korchynskyi O., Makarov S.S., Maixner W., Diatchenko L. (2006). Human catechol-O-methyltransferase haplotypes modulate protein expression by altering mRNA secondary structure. Science.

[31] Duan J., Wainwright M.S., Comeron J.M., Saitou N., Sanders A.R., Gelernter J., Gejman P.V. (2003). Synonymous mutations in the human dopamine receptor D2 (*DRD2*) affect mRNA stability and synthesis of the receptor. Hum. Mol. Genet..

[32] Gotea V., Gartner J.J., Qutob N., Elnitski L., Samuels Y. (2015). The functional relevance of somatic synonymous mutations in melanoma and other cancers. Pigment Cell Melanoma Res..

