# Supplementary figures and images for: Variation in a Newly Identified Caprine KRTAP Gene Is Associated with Raw Cashmere Fiber Weight in Longdong Cashmere Goats
Source: Genes (Basel). 2021 Apr 22;12(5):625. doi: 10.3390/genes12050625 (PMC8143586; doi:10.3390/genes12050625)

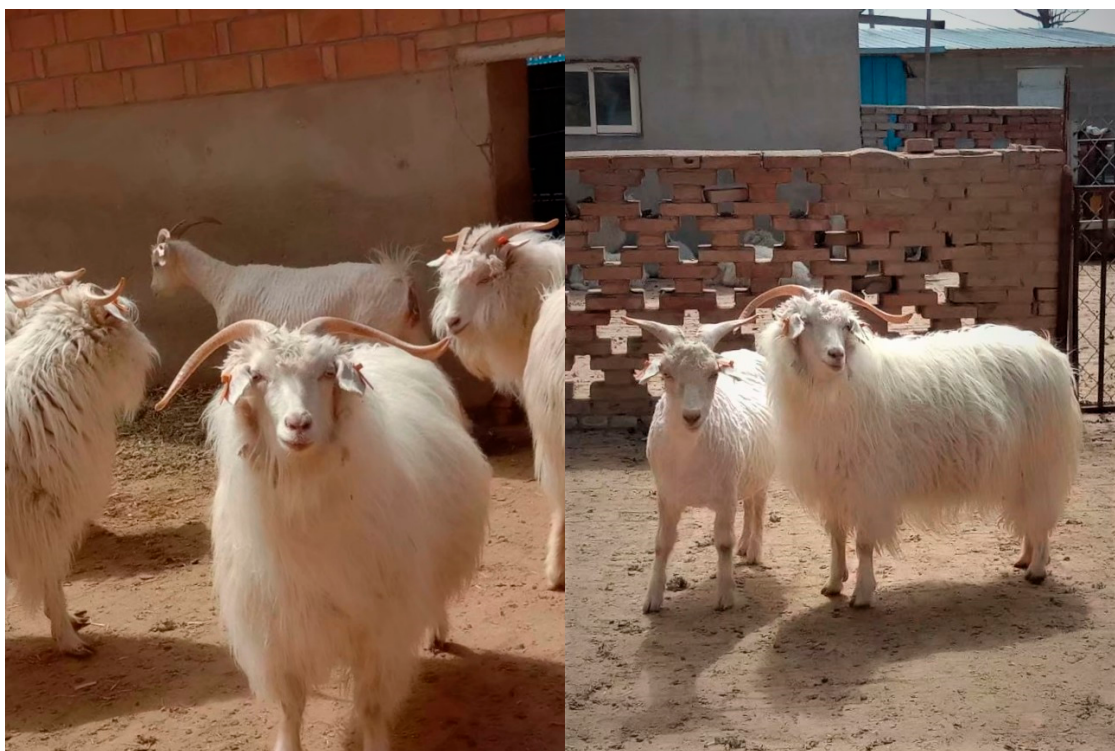

**Supplementary Figure S1.** Adult and juvenile Longdong cashmere goats

Supplement: Supplementary file 1 [file genes-12-00625-s001.zip › genes-1165397-supplementary.pdf]
